# Deletion of DGCR8 in VSMCs of adult mice results in loss of vascular reactivity, reduced blood pressure and neointima formation

**DOI:** 10.1038/s41598-018-19660-z

**Published:** 2018-01-23

**Authors:** Yanan Zou, Zixuan Chen, Brett L. Jennings, Guannan Zhao, Qingqing Gu, Anindya Bhattacharya, Yan Cui, Bo Yu, Kafait U. Malik, Junming Yue

**Affiliations:** 1 Department of Cardiology, The Second Affiliated Hospital of Harbin Medical University, Harbin, 150086 P.R. China; 2 Departments of Pathology, The University of Tennessee Health Science Center, 19 S. Manassas St., Memphis, TN 38163 USA; 3 Departments of Pharmacology, The University of Tennessee Health Science Center, 19 S. Manassas St., Memphis, TN 38163 USA; 4 Department of Genetics Genomics and Informatics, The University of Tennessee Health Science Center, 19 S. Manassas St., Memphis, TN 38163 USA; 5 School of Life Science, Beijing Institute of Technology, Beijing, P.R. China

## Abstract

DiGeorge syndrome chromosomal region 8 (DGCR8), a double-stranded-RNA-binding protein, participates in the miRNA biogenesis pathway and contributes to miRNA maturation by interacting with the RNAase III enzyme Drosha in cell nuclei. To investigate the role of DGCR8 in vascular smooth muscle cells (VSMCs) at the postnatal stages, we generated tamoxifen-inducible VSMC specific knockout (iKO) mice by crossing DGCR8^loxp/loxp^ with VSMC specific tamoxifen-inducible Cre transgenic mice SMA-Cre-ER^T2^. DGCR8iKO mice display reduced body weight one month following tamoxifen treatment and died around 3 months. Blood pressure and vascular reactivity were significantly reduced in DGCR8iKO mice compared to control. Furthermore, loss of DGCR8 in VSMCs inhibited cell proliferation, migration and neointima formation. VSMC differentiation marker genes, including SMA and SM22, were downregulated in DGCR8 iKO mice. The majority of miRNAs were downregulated in DGCR8iKO mice. Disruption of the DGCR8-mediated miRNA biogenesis pathway attenuated multiple signaling pathways including ERK1/2 and AKT. Our results demonstrate that the DGCR8-mediated miRNA pathway is required for maintaining blood pressure, vascular reactivity and vascular wall remodeling at the postnatal stages.

## Introduction

DiGeorge syndrome chromosomal region 8 (DGCR8), a double-stranded RNA binding protein, participates in the miRNA biogenesis pathway by interacting with the RNase III enzyme Drosha and forming a microprocessor in the cell nucleus that processes primary miRNA (pri-miRNA) into precursor miRNA (pre-miRNA)^1–3^. Pre-miRNAs are subsequently transported into the cytoplasm and are cleaved by RNAase III enzyme Dicer into the 22 nucleotides of mature miRNAs, through RNA-induced silencing complex (RISC) containing Dicer and Ago2, thus suppressing protein translation at the posttranscriptional level^4–6^. miRNAs play robust roles in maintaining vascular smooth muscle cell (VSMC) function by regulating VSMC proliferation and differentiation. The miR-17/92 cluster promotes VSMC proliferation and differentiation^7^. However, several miRNAs have been identified that regulate VSMC phenotypic switches. Thus, miR-663 inhibits PDGF-BB-induced cell proliferation and migration, whereas it promotes VSMC differentiation marker gene expression including SMA, SM22, CNN1 and MYH11^8^. In addition, miR-195, miR-143/145, and miR-133 were identified and characterized as regulating the VSMC phenotypic switch^9–11^. These studies indicate that miRNAs may play different roles in contributing to VSMC functions by regulating VSMC proliferation, migration, and differentiation.

To investigate the global function of miRNA in VSMCs, we have generated Drosha, DGCR8 and Dicer VSMC-specific conditional knockout (cKO) mice by disrupting the miRNA biogenesis pathway. VSMC-specific DGCR8, Drosha, and Dicer cKO mice died during embryonic stages and all cKO mice share similar phenotypes, including extensive hemorrhaging in the liver and dilated vascular wall. Dicer and DGCR8 cKO mice display developmental delay while Drosha cKO did not^12^. DGCR8 cKO mice died several days earlier than Drosha and Dicer cKO mice^7,12,13^. miRNA expression was downregulated in DGCR8, Drosha, and Dicer cKO mice compared to control mice, although those downregulated miRNAs are not exactly the same miRNAs among those cKO mice. In addition, a slightly different phenotype was reported in Dicer VSMC cKO mice by deleting exons 20–21 in VSMCs. Indeed, these Dicer cKO mice didn’t display growth delay between cKO and control mice^14^. Since DGCR8cKO mice display a more severe phenotype than that of Drosha or Dicer cKO, the DGCR8-dependent miRNA biogenesis pathway may play a more important role than Drosha or Dicer in regulating VSMC function^13,14^. Moreover, Dicer processed not only miRNA but also small interfering RNA (siRNA)^15–17^. Some miRNA maturation, such as miR-451, does not require Dicer, but is Ago2-dependent^18–21^, indicating that Dicer is not specific for miRNA maturation. However, DGCR8 primarily targets miRNA maturation as demonstrated by previous studies^22^.

To further address how DGCR8-dependent miRNA biogenesis pathways control VSMC function at the postnatal stages including blood pressure and vascular reactivity, we have generated VSMC–specific, tamoxifen-inducible KO (iKO) mice by crossing DGCR8^loxp/loxp^ with SMA-Cre-ER^T2^ mice^23^. DGCR8iKO mice display reduced blood pressure, vascular reactivity, dilated vascular wall, and died around three months following tamoxifen injection. Loss of DGCR8 in VSMCs leads to reduced cell proliferation and migration. VSMC marker genes SMA, SM22 and MYH11 were significantly reduced in DGCR8iKO mice compared to controls. The majority of miRNAs were downregulated and multiple signaling pathways were dysregulated, including two attenuated cellular survival pathways ERK1/2 and AKT in DGCR8iKO mice.

## Results

### Inducible deletion of DGCR8 in VSMCs of adult mice leads to postnatal death

**Figure 1:**
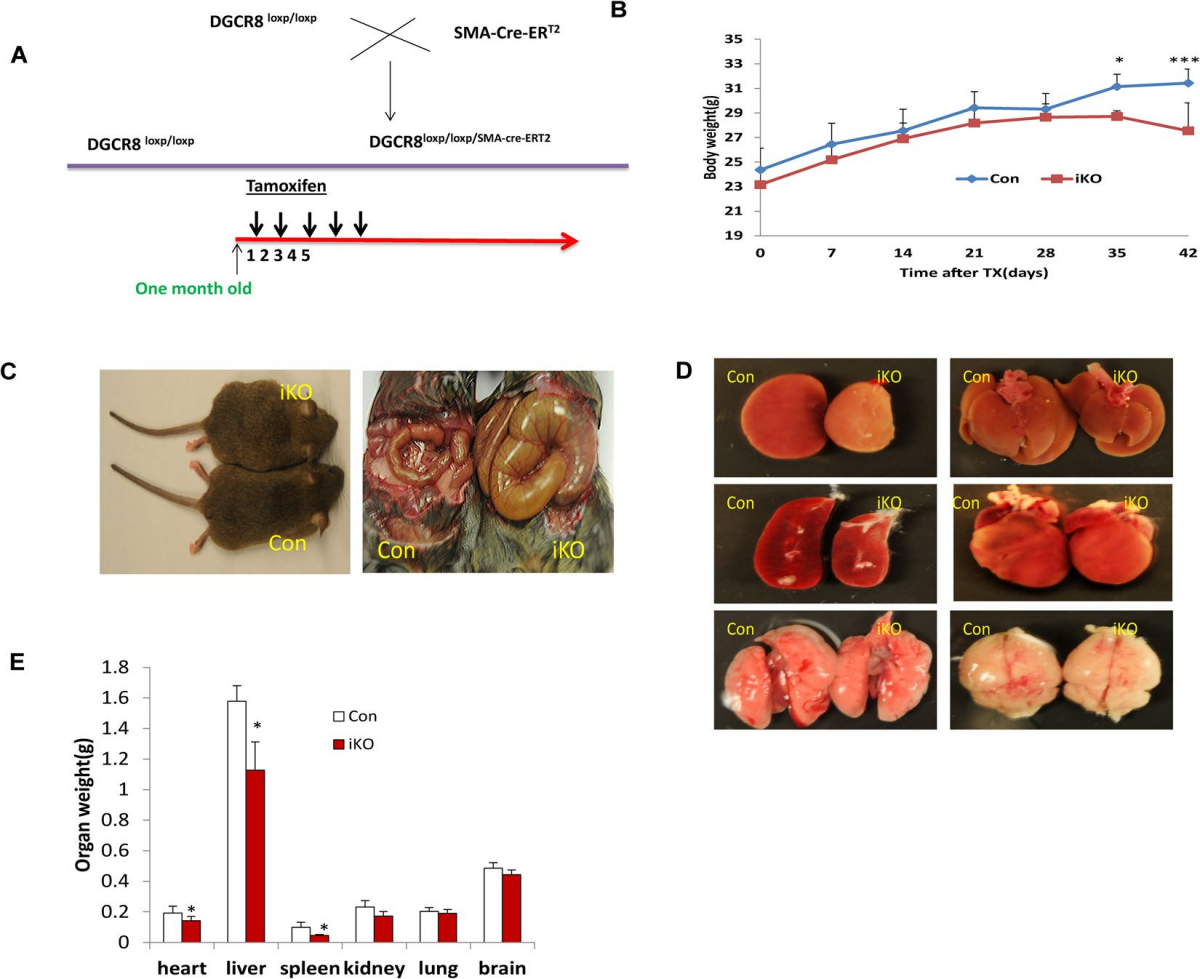
Tamoxifen inducible deletion of DGCR8 in VSMCs of adult mice. (**A**) Schematic diagram of generating a VSMC-specific tamoxifen-inducible knockout (iKO) mice by crossing DGCR8^loxp/loxp^ with SMA-Cre-ER^T2^ mice. DGCR8iKO (DGCR8^loxp/loxp; SMA-Cre-ERT2^) and control mice (DGCR8^loxp/loxp^) were injected with tamoxifen for five days consecutively when they were one month old. (**B**) The DGCR8iKO mice displayed significant weight loss after one month following tamoxifen injection (*p < 0.05;***p < 0.01, n = 6). (**C**) DGCR8iKO mice displayed a distended cecum compared to control mice at two and half months following tamoxifen injection. (**D** and **E**) Organ morphologies in DGCR8iKO and control mice at two and half months old. Significant organ differences were found in heart, liver and spleen (*p < 0.05).

DGCR8 deletion in VSMCs was achieved by intraperitoneally injecting tamoxifen into DGCR8^loxp/loxp; SMA-Cre-ERT2^ for five consecutive days. DGCR8^loxp/loxp^ mice were injected with tamoxifen or DGCR8^loxp/loxp^ mice were injected with Sunflower oil as our control mice (Fig. 1A). We found that the body weight of DGCR8iKO mice was significantly reduced compared to control mice one month following tamoxifen injection, although we didn’t observe a significant difference during the first month (Fig. 1B). DGCR8iKO mice died three months following tamoxifen injection. (Fig. 1C). We observed that the sizes of heart, liver and spleen were significantly smaller in DGCR8iKO than that in control mice, although no significant differences were found in the kidney, lung and brain when they were two and half months old (Fig. 1D,E). There were no obvious histological abnormality observed by examining H.E stained sections among those tissues.

### Loss of DGCR8 in VSMCs resulted in reduced blood pressure and vascular reactivity

**Figure 2:**
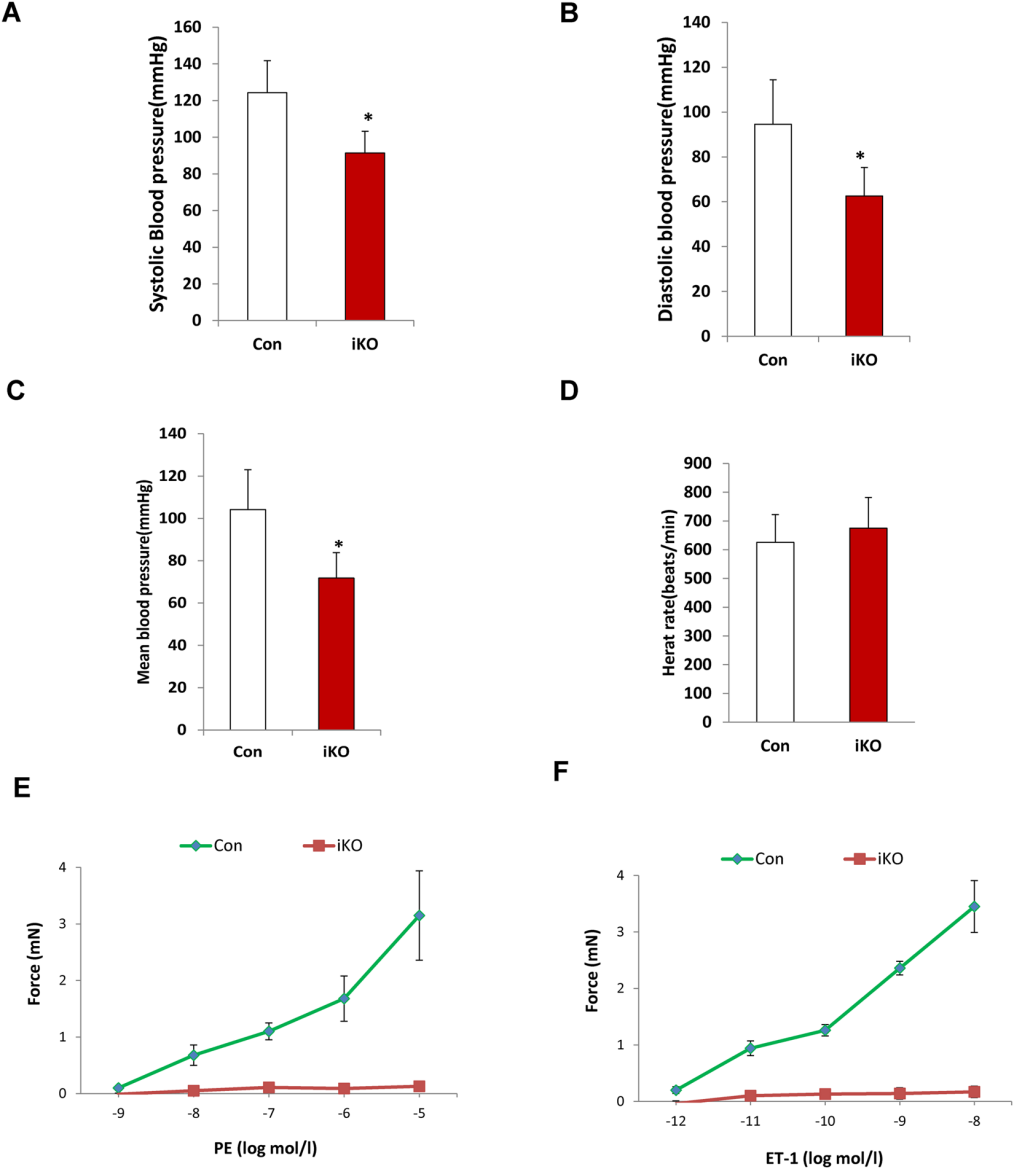
DGCR8iKO mice displayed reduced blood pressure and vascular reactivity. (**A,B,C**) Blood pressure was measured in DGCR8iKO and control mice one month after tamoxifen injection using tail-cuff method. DGCR8iKO mice displayed significantly reduced blood pressure including systolic (**A**), diastolic (**B**) and mean (**C**) compared to controls (N = 5 per group, *p < 0.05). (**D**) DGCR8iKO mice didn’t display significant differences in heart rate compared to controls. (**E** and **F**) The vascular reactivity was measured in thoracic aorta isolated from DGCR8iKO and control mice using a wire myography. DGCR8iKO mice displayed diminished vascular response to PE (***p < 0.001) and ET-1 (***p < 0.001), respectively.

DGCR8iKO mice are developmentally normal during first month following tamoxifen injection compared to controls. To examine whether loss of DGCR8 in VSMCs leads to vascular functional alterations, we measured blood pressure at one month following tamoxifen injection using tail-cuff method. SBP, DBP, and MAP were significantly reduced in DGCR8iKO mice compared to controls (Fig. 2A,B and C). However, there was no significant difference on heart rate between DGCR8iKO and control mice (Fig. 2D). To further determine the cause of low blood pressure in DGCR8iKO mice, we measured the vascular reactivity of thoracic aorta using wire myography. The vascular reactivity, as demonstrated by the response to vasoconstrictors phenylephrine (PE) and endothelin-1 (ET-1), was completely abolished in DGCR8iKO mice compared to control mice (Fig. 2E,F).

### Disruption of DGCR8 in VSMCs resulted in reduced proliferation, migration, and neointimal formation in wire-injured carotid mouse artery

**Figure 3:**
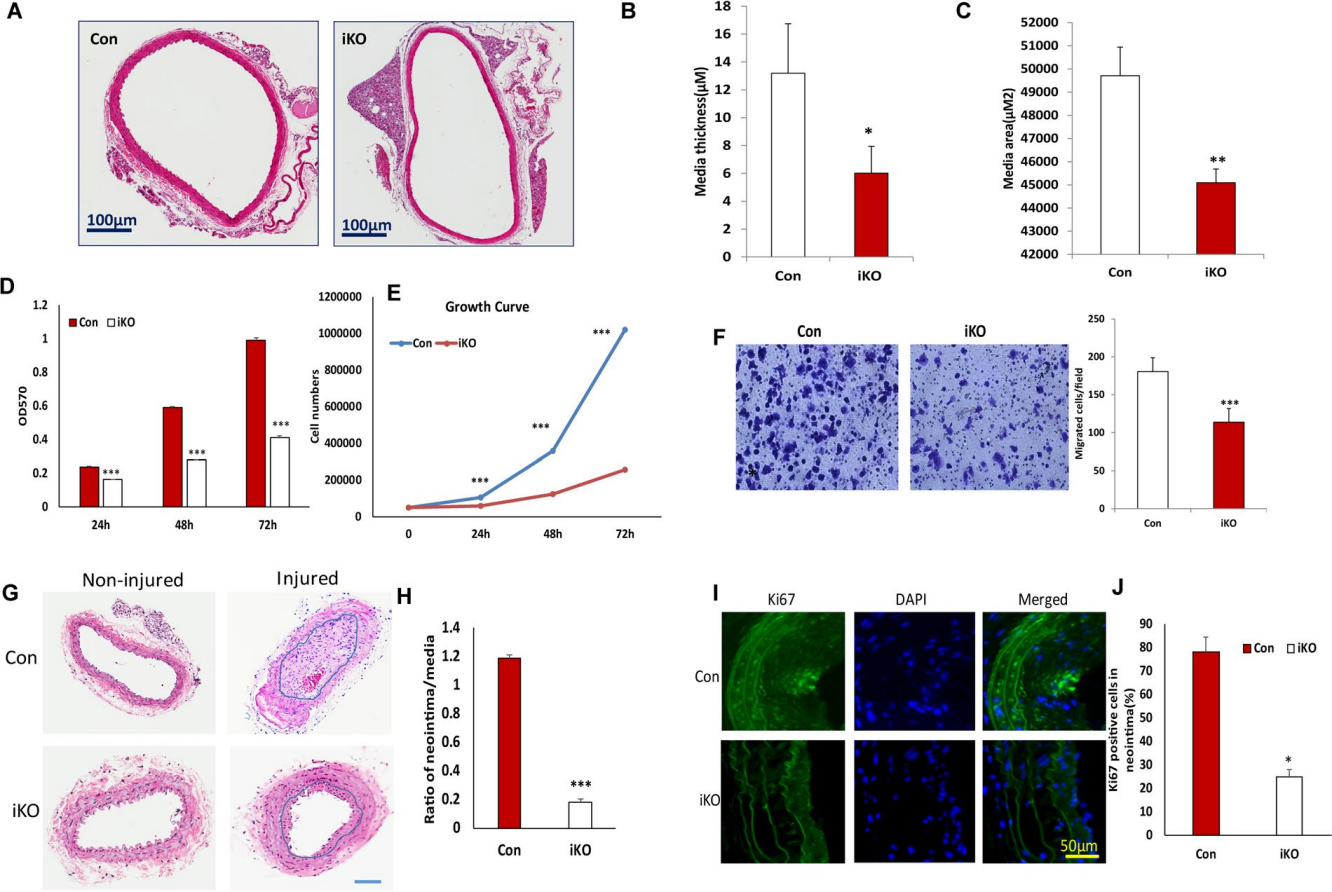
Loss of DGCR8 in VSMCs significantly reduced cell proliferation and migration *in vitro* and neointima formation *in vivo*. (**A**) Sections of thoracic aorta from DGCR8iKO and control mice were stained with hematoxylin and eosin. (**B** and **C**), The medial area and thickness of thoracic aorta of DGCR8iKO and controls were measured using a Nikon microscopy program for three sections from each mouse, respectively. Scale Bars indicate 100 µm (*p < 0.05; **p < 0.01). (**D** and **E**) Cell proliferation in primary VSMCs isolated from DGCR8iKO and control mice were measured using MTT (**E**) and cell counting (**F**). Data were analyzed using ANOVA and post-hoc least significance (*p < 0.05; *p < 0.01). (**F**) Cell migration in primary VSMCs of DGCR8iKO and control mice was measured using transwell plate and stained with crystal violet (***p < 0.001) (**G**) Representative cross-sections of uninjured (**A**,**D**) and injured (**B**,**C**)carotid arteries stained with hematoxylin and eosin from DGCR8iKO and control mice 28 days following injury. (**H**) The ratio of neointima to media area. Data were analyzed with t-test (***p < 0.001). N indicates number of mice. I: Immunofluorescent staining of VSMC proliferation marker Ki67 (green) in a representative cross-section of injured carotid arteries of DGCR8iKO and control mice. Cell nuclei were counterstained with DAPI. J: A quantification of Ki67 positive cells were in the neointima was performed from 6 cross-sections of 3mice. Data were analyzed with t-test and shown in mean ± SD (*p < 0.05).

To examine whether loss of DGCR8 in VSMCs leads to vascular wall remodeling, thoracic aorta were sectioned and stained with H&E. The media area and thickness of thoracic aorta in DGCR8 were significantly reduced in DGCR8iKO mice compared to controls (Fig. 3A,B and C). To further assess whether loss of DGCR8 reduced cell proliferation, we examined the proliferation of primary VSMCs isolated from DGCR8iKO and control mice using MTT and cell counting for three days. Proliferation of VSMC isolated from DGCR8iKO mice was significantly reduced compared to that obtained from control mice as determined by both methods (Fig. 3D,E). Cell migration was detected by transwell plate, and loss of DGCR8 in VSMCs also caused significant reduction of migrated cells (Fig. 3F). To analyze the impact of DGCR8 on VSMC proliferation and migration *in vivo*, we performed wire-guided carotid artery injury in DGCR8 iKO and control mice. Loss of DGCR8 in VSMCs led to significantly reduced neointima formation (Fig. 3G,H). To examine the VSMC proliferation *in vivo*, we performed immunofluorescent staining using antibodies against cell proliferation marker Ki67, which showed significant reduction in DGCR8iKO compared to control mice (Fig. 3I,J).

### DGCR8 iKO mice displayed reduced VSMC marker gene expression

**Figure 4:**
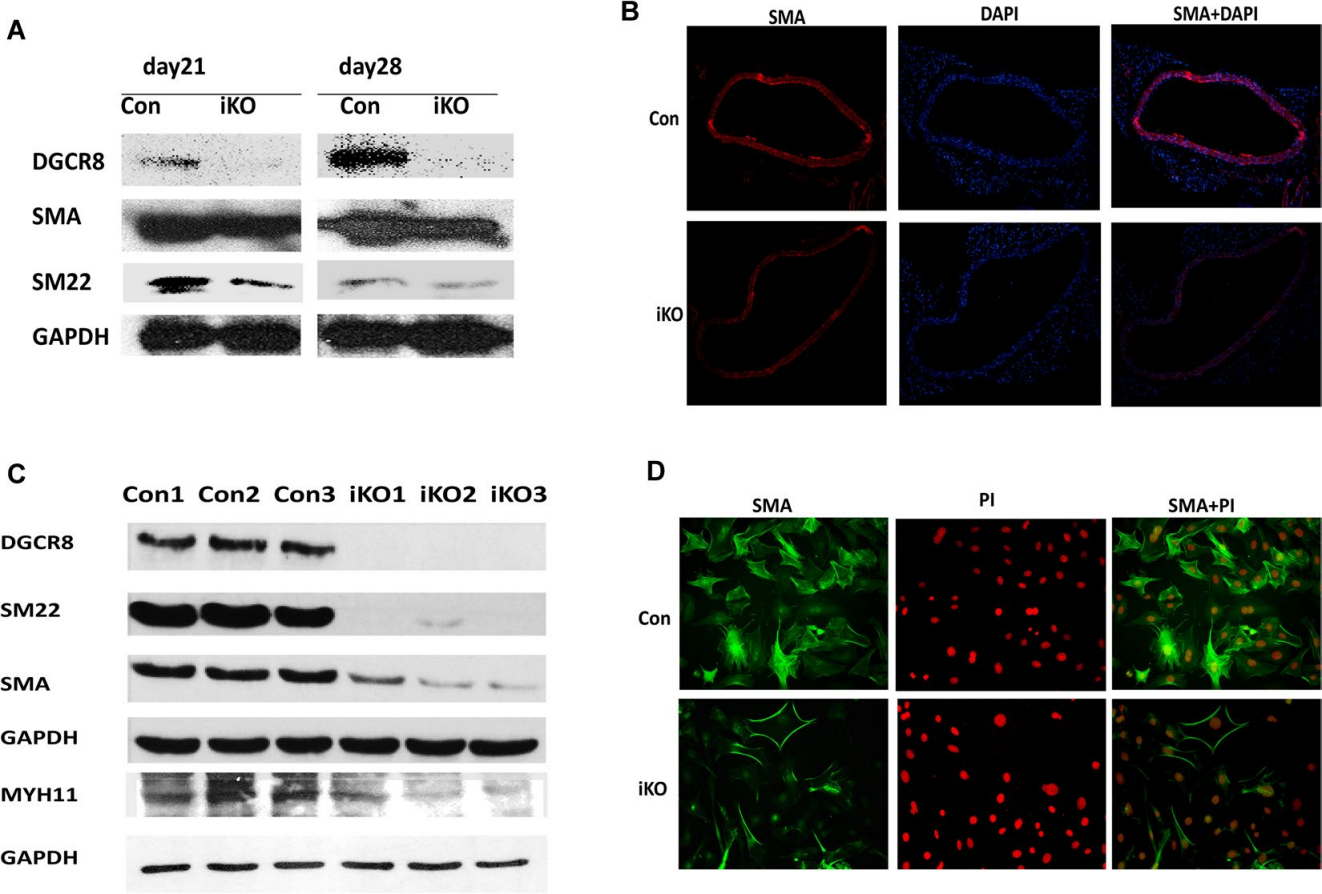
DGCR8iKO mice display reduced VSMC differentiation marker gene expression. (**A**) SMA and SM22 were detected from DGCR8iKO and control mice on day 21 and day 28 following tamoxifen injection by Western blot (full blot shown in Figure S1). (**B**) Sections of thoracic aorta of DGCR8iKO and control mice on day 28 following tamoxifen injection were detected by SMA immunostaining. SMA was stained with red immunofluorescence and nuclei were stained blue with DAPI. (**C**) VSMC marker gene expression in primary VSMCs isolated from three different DGCR8iKO and control mice was detected by Western blot (full blot shown in Fig. S2A,B and C). (**D**) SMA expression in primary VSMCs of DGCR8iKO and control mice was immunostained with SMA antibody. Cell nuclei were stained by PI.

To determine whether loss of DGCR8 affects SMC differentiation in adult mice, we examined expression of SMA and SM22 in DGCR8iKO and control mice at days 21 and 28 following tamoxifen injection. SMA and SM22 were significantly downregulated in DGCR8iKO mice compared to control mice. DGCR8 expression was diminished in DGCR8iKO mice compared to controls (Fig. 4A). In addition, SMA expression in thoracic aorta was detected by immunofluorescent staining and it was downregulated in aorta of DGCR8iKO compared to controls (Fig. 4B). We also detected VSMC differentiation marker gene expression in the primary VSMCs isolated from DGCR8iKO and control mice, and SMA, SM22 and MYH11 were downregulated as shown by Western blot (Fig. 4C). SMA expression was also downregulated in primary VSMCs of DGCR8iKO compared to controls, as shown by immunostaining (Fig. 4D).

### Disruption of DGCR8-dependent miRNA biogenesis pathway in VSMCs of adult mice dysregulates miRNA production and multiple signaling pathways

**Figure 5:**
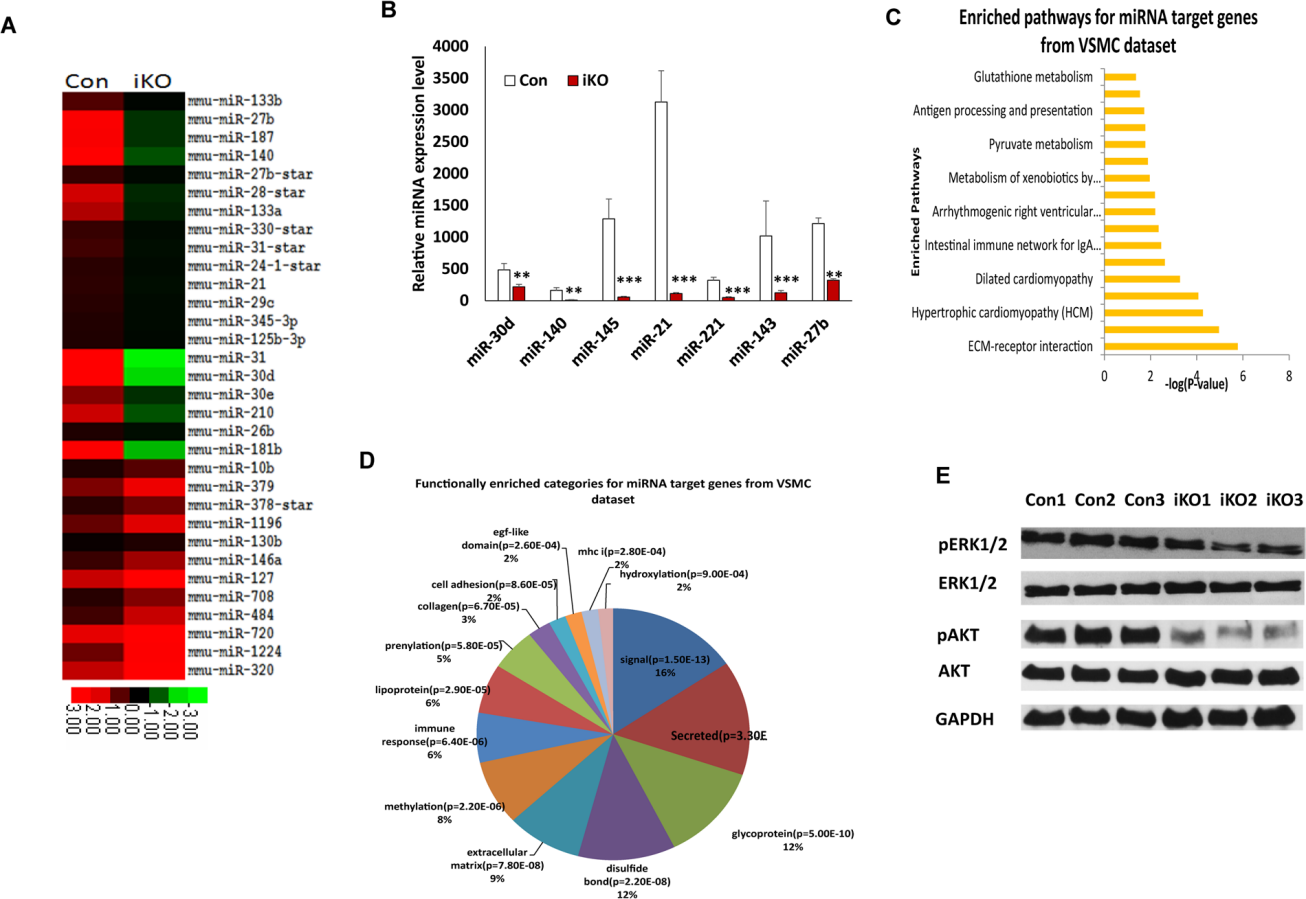
miRNA and multiple signaling pathway were dysregulated in DGCR8iKO mice. (**A**) Heat map of miRNA expression in DGCR8iKO and control mice. (**B**) miRNA expression was verified by polyA tailing real-time RT-PCR. All of them were downregulated at least several-fold in DGCR8iKO compared to control mice (**p < 0.01; ***p < 0.001). (**C**) miRNA-targeted multiple gene pathways were altered in DGCR8iKO mice as analyzed using DAVID bioinformatics. (**D**) miRNA-targeted genes were functionally enriched. (**E**) Two cellular survival pathways in the primary VSMCs of DGCR8iKO were examined by Western blot (full blot shown in Fig. S2A and B).

DGCR8 is a key component of microprocessor in the miRNA biogenesis pathway and contributes to miRNA production by facilitating the cleavage of Drosha from pri-miRNA to pre-miRNA. To examine how loss of DGCR8 in VSMCs of adult mice affects the miRNA expression, we performed a miRNA array by extracting RNA from thoracic aorta of DGCR8iKO and control mice one month after tamoxifen injection. There were 232 miRNAs that were downregulated and 20 were upregulated in the aortas of DGCR8iKO mice compared to controls, and 32 miRNAs, over two-fold up or downregulated, are displayed in the heat map (Fig. 5A). Several miRNAs were selected and validated by polyA tailing real-time RT-PCR. Both miR-143 and miR-145 are well-studied in regulating VSMC differentiation^11,24–26^. In DGCR8iKO mutants, miR-143 and 145 were downregulated, ~8 and 22-fold compared with controls, respectively. The miR-221/222 cluster regulates neovascularization by targeting the signal transducer and activator of transcription 5A (stat5A), and is associated with coronary artery disease^27^. miR-221 was 6-fold downregulated in DGCR8iKO mice. miR-21, a miRNA implicated in vascular disease^28^, was approximately 27-fold downregulated (Fig. 5B). miR-30d was found to promote cardiomyocyte growth and inhibited cell apoptosis, and downregulated miRNAs in DGCR8iKO mice by ~3 fold; however, miR-30d has not yet been investigated in VSMCs. miR-27b contributed to cardiac hypertrophy and reduced ~6 fold in DGCR8iKO mice compared to controls.

Target genes of 32 miRNAs up/down regulated by at least two fold were computationally determined using TargetScan^29^. The target gene lists were further analyzed for functional enrichment using the Database for Annotation, Visualization and Integrated Discovery (DAVID)^30^. Figure 5C shows the cell signaling pathways that were enriched among the target genes (adjusted p-value < 0.01 using the Benjamini-Hochberg procedure). Multiple signaling pathways were dysregulated in DGCR8iKO mice including the cardiac hypertrophic pathway (Fig. 5C). Figure 5D shows the gene ontology (GO) categories that were enriched among the miRNA target genes (adjusted p-value < 0.01 using the Benjamini-Hochberg procedure). Two cellular survival pathways ERK1/2 and AKT were attenuated in DGCR8iKO mice compared to controls as shown by Western blot (Fig. 5E).

## Discussion

In this study, we have revealed that tamoxifen-mediated inducible deletion of DGCR8 in VSMCs of adult mice led to death around three months, although there were no obvious phenotypes during the first month following tamoxifen injection. DGCR8iKO mice displayed dilated gastrointestinal track, especially distended cecum, indicating that loss of DGCR8 in SMCs of adult mice leads to reduced contraction in the digestive system, so gastrointestinal dysfunction is likely the cause of death of DGCR8iKO mice. Loss of Dicer in SMCs of postnatal stages resulted in a phenotype similar to that observed in DGCR8iKO mice, while SMC-specific myosin heavy chain (Myh11) promoter-driven Cre mice were used in both studies^31,32^. DGCR8iKO mice displayed reduced blood pressure, and vascular contractility as the response to vasoconstrictor PE and ET1. In addition, DGCR8iKO mice also showed the significantly reduced neointima formation compared to control mice following wire-induced carotid artery injury. Our data indicate that DGCR8 was required for vascular wall remodeling at the postnatal stages. Disruption of DGCR8 in VSMCs led to reduced blood pressure, which was also reported in VSMC-specific Dicer iKO mice^32^. Low blood pressure in DGCR8iKO mice was most likely due to decreased vascular contraction, as we showed that DGCR8iKO mice lost vascular reactivity to treatment with vasoconstrictors PE and ET-1.

We have previously shown that conditional knockout (cKO) of DGCR8 in VSMCs leads to embryonic lethality in mice because of disrupted vasculature during embryonic development^7^. However, DGCR8iKO mice died around three months following tamoxifen injection, which was also observed in Dicer iKO mice^32^. These studies indicated that DGCR8 plays a similar role in vascular development. The phenotypic similarity between DGCR8iKO and Dicer iKO mice suggests that both genes may contribute to SMC function by participating in the miRNA biogenesis pathway. DGCR8 functions upstream of miRNA biogenesis pathway to facilitate Drosha cleavage of pri-miRNA into pre-miRNAs, whereas Dicer is downstream by cleaving pre-miRNA into mature miRNAs. Deletion of DGCR8 in VSMCs disrupts miRNA maturation at earlier stages than Dicer does. In addition to miRNAs, DGCR8 has been shown to regulate other mRNAs, short and long non-coding RNAs, which is independent of Drosha cleavage^33^. Further studies are required to understand how DGCR8 interacts with other mRNAs and noncoding small or long noncoding RNAs to regulate VSMC function.

We have also previously shown that DGCR8 promotes VSMC proliferation and differentiation by characterizing VSMC-specific DGCR8cKO mice^7^. Similarly, loss of DGCR8 in VSMCs of adult mice resulted in reduced cell proliferation and differentiation (Figs 3 and 4). Cell migration was also significantly reduced in primary VSMCs of DGCR8iKO compared to control mice. DGCR8 may contribute to VSMC function by regulating cell proliferation, migration and differentiation through a gene regulatory network, including miRNAs or other regulated genes. Based on our miRNA array data, the majority of miRNA expressions were downregulated whereas some miRNAs were upregulated in VSMCs of DGCR8 compared to controls. Those upregulated miRNAs may not fully follow the conventional miRNA biogenesis pathway. However, individual miRNA may function differently in regulating VSMC phenotypes. The miR-143/145 cluster contributes to phenotype switch by inhibiting cell proliferation and promoting differentiation. However, we showed that the miR-17/92 cluster promotes VSMC proliferation and differentiation^7^. Therefore, DGCR8 regulates VSMC function through a gene/miRNA network. Using a bioinformatics approach, we analyzed the signaling pathway through miRNA targeted genes. There were multiple signaling pathways deregulated in DGCR8iKO mice, including hypertrophic cardiomyopathy (Fig. 5B). We have verified that two cardiovascular pathways ERK1/2 and AKT were attenuated in primary VSMCs of DGCR8iKO compared to control mice, which was consistent with what we have found in VSMC-specific DGCR8cKO mice^7^. However, both pathways were not altered in VSMCs specific Dicer cKO mice^14^. So far, it is not clear why both pathways were not altered in Dicer cKO mice, since both DGCR8 and Dicer contribute to miRNA maturation. However, the attenuation of both pathways suggest that DGCR8 may play a more important role in VSMC survival than Dicer. Both pathways may be regulated through miRNAs. For example, miR-21 targets PTEN and Sprouty2, thus regulating downstream AKT and ERK1/2 pathways, respectively. miR-21 was significantly reduced in VSMCs of DGCR8iKO compared to control mice. However, either DGCR8 or Dicer interacts with other genes or small or long non-coding RNAs in addition to miRNA maturation. Therefore, DGCR8 regulates multiple signaling pathways through miRNA-regulated genes, as shown in this study. Further studies are required to understand how DGCR8 contributes to VSMC function by regulating cell proliferation, migration, and differentiation.

## Materials and Methods

All methods were performed in accordance with the relevant guidelines and regulations.

### Cell culture

Primary VSMCs were isolated from aorta of DGCR8iKO and control mice, one month following tamoxifen injection. The aorta was isolated by removing the surrounding fat tissues and washed in HBSS media and then left in an enzyme solution (0.125 mg/ml elastase, 0.250 mg/ml soybean trypsin inhibitor, and 1.0 mg/ml collagenase) at 37 °C in a shaker bath for 30–45 minutes. The adventitial layer was removed and the aorta was minced into small pieces and incubated in an enzyme solution for 2.5 h. Cells were centrifuged and suspended in 10% DMEM for culture.

### Generation of VSMC-specific Tamoxifen-induced DGCR8 KO (iKO) mice

DGCR8^loxp/loxp^ mice were a kind gift from Dr. Elaine Fuchs, Rockefeller University^22^. SMA-Cre-ER^T2^ transgenic mice were obtained from Dr. Pierre Chambon and Dr. Daniel Metzger, France^23^. To generate the VSMC specific DGCR8iKO mice, we crossed DGCR8^loxp/loxp^ with SMA-Cre-ER^T2^ transgenic mice to generate DGCR8^loxp/+; SMA-Cre-ERT2^ mice and then intercrossed or bred with DGCR8^loxp/loxp^ mice to generate DGCR8^loxp/loxp; SMA-Cre-ERT2^ mice. For all experiments, DGCR8^loxp/loxp; SMA-Cre-ERT2^ mice were treated with tamoxifen as the experimental group (DGCR8iKO mice). DGCR8^loxp/loxp^ mice were treated with tamoxifen or DGCR8iKO mice were treated with vehicle (Sunflower oil) as our control group. DGCR8iKO mice were induced to delete DGCR8 in VSMCs of adult mice when they were one month old and injected with tamoxifen (50 mg/Kg/day) for five consecutive days. All mice were on a mixed C57BL/6/129 background. Sex-matched mice were used for most of the experiments, except that male mice were used for blood pressure experiments. Animal experiments were approved by the Institutional Animal Care and Use Committee at the University of Tennessee Health Science Center.

### Genotyping

DGCR8iKO and control mice were genotyped by PCR using the same primers for VSMC-specific DGCR8cKO mice and SMA-Cre-ER^T2^ mice were genotyped using Cre-specific primers as described^7^.

### Blood Pressure Measurement

Blood pressure was measured using tail-cuff method (Kent Scientific, Torrington, CT; model XBP 1000) as described previously^34^. Briefly, male DGCR8iKO or control mice were acclimatized to the experimental conditions in a restricted chamber for a week prior to recording the blood pressure. Systolic blood pressure (SBP), diastolic blood pressure (DBP), mean arterial pressure (MAP), and heart rate were collected for each mouse.

### Measurement of vascular reactivity

The aorta were cleaned of surrounding tissue, and ∼2-mm rings were mounted in a wire myograph system (model 610 M, Danish Myo Technology, Aarhus, Denmark). Vessels were continuously bathed in Krebs buffer (in mmol/l: 118 NaCl, 4.7 KCl, 25 NaHCO_3_, 1.2 MgSO_4_, 1.2 KH_2_PO_4_, 11.1 glucose, and 2.5 CaCl_2_·2H_2_O) at 37 °C, which was gassed with 95% O_2_-5% CO_2_ to maintain the pH at 7.4, and were allowed to equilibrate for ∼30 min. To confirm the viability of the vessels, they were initially tested for constriction to 60 mmol/l KCl and then washed three times with fresh Krebs buffer. Cumulative concentration-response curves to phenylephrine (PE) and endothelin-1 (ET-1) were obtained, and responses were measured as the force of contraction (in mN).

### Wire-guided mouse carotid artery injury

DGCR8iKO and control mice were generated following tamoxifen injection for five consecutive days at one month old and the wire-guided carotid artery injury was performed one day after the last injection. Mice were anesthetized by intraperitoneally injecting ketamine/Xylazine (80/5 mg/Kg body weight). The right carotid artery was exposed via a midline neck incision. The common, external, and internal carotid arteries were identified and isolated (using 7.0 non-absorbable surgical suture). A 30-gauge needle was employed to make an arteriotomy at the external carotid. A fixed straight core guide wire (Cook Incorporated, Bloomington, IN) was introduced into the common carotid artery and rotated five times. The external carotid artery was ligated, blood flow to the common carotid artery was restored, and the skin was closed using 6-0 Nylon suture black monofilament (Ethilon). Buprenorphine SR (1–1.2 mg/kg) was injected to relieve pain following surgery. Mice were sacrificed at 28 days following injury and carotid arteries from both sides were collected for histological analysis.

### Histological Analysis

To characterize the phenotype of DGCR8iKO mice, aorta were collected and fixed in 10% formalin and embedded in paraffin, sectioned, and stained with H&E.

### miRNA Array

To determine the expression of miRNAs in VSMCs of DGCR8iKO and control mice, total RNA was isolated from aorta one month after tamoxifen injection using TRIzol reagent (Invitrogen), and RNA was further purified with the RNeasy MinElute cleanup kit (Qiagen, Valencia, CA). The miRNA microarray profiling was performed using Affymetrix GeneChip miRNA array 3.0 (Santa Clara, CA) as described^7^.

### Detection of miRNA expression using PolyA tailing real-time RT-PCR

Total RNA was extracted from primary VSMCs isolated from DGCR8iKO and control mice. Poly(A) tailing real-time RT-PCR was performed on a LightCycler 4800 real-time PCR instrument (Roche) as described previously^35^. A melting curve analysis was performed to examine the PCR product specificity. The relative miRNA expression was calculated using the Δ_2_Ct method and expressed as mean ± S.D. by normalizing to human U6 small nuclear RNA.

### Immunofluorescence

Deparaffinized sections were rehydrated, and the antigen was retrieved by incubation of the slides for 30 min at 95–100 °C in 10 mm sodium citrate, 0.05% Tween 20 (pH 6.0). The sections were treated with blocking buffer (5% normal goat serum, 3% bovine serum albumin, and 0.1% Triton X-100 in PBS) for 1 h. To detect the VSMC proliferation and differentiation, sections were incubated with primary antibody to cell proliferation marker (Ki67) or α-smooth muscle actin (αSMA) at 4 °C overnight, respectively. After three rinses for 5 min with 0.05% Tween 20 in PBS, the sections were incubated with Alexa Fluor 488- or Alexa Fluor 594-conjugated goat anti-rabbit or mouse secondary antibody (Invitrogen, 1:200 in Tween 20 in PBS) for 1 h at room temperature. After three washes, the sections were mounted with Vectashield medium containing DAPI or propidium iodide (Vector Laboratories, Inc., Burlingame, CA).

### Western Blotting

Thoracic aorta were collected from DGCR8iKO or control mice and sonicated in lysis buffer (Thermo Scientific, Rockford, IL) containing 1% Halt proteinase inhibitor mixture (Thermo Scientific). Primary VSMCs isolated from DGCR8iKO and control mice were also collected to detect the expression of DGCR8 or VSMC marker gene expression. Approximately 80 µg protein for each sample was loaded on 8% SDS-PAGE gels and transferred to nitrocellulose membranes, which was then blocked with 5% nonfat milk for 1 h and incubated with primary antibodies against DGCR8, MYH11, Ki67, SM22 (Santa Cruz Biotechnology, Santa Cruz, CA), β-actin, SMA, and GAPDH (Sigma), phospho(p)-ERK1/2, phosphor (p)-AKT, ERK1/2, AKT (Cell Signaling Technology, Danvers, MA).

### Statistical Analysis

Data shown are the mean ± S.D. from at least three different experiments. The differences were analyzed using Student’s ttest or ANOVA and post-hoc least significant difference. *p* values < 0.05 were considered significant.

## Electronic supplementary material

Supplementary information
