# Supplementary material for: Deletion of DGCR8 in VSMCs of adult mice results in loss of vascular reactivity, reduced blood pressure and neointima formation
Source: Sci Rep. 2018 Jan 23;8:1468. doi: 10.1038/s41598-018-19660-z (PMC5780492; doi:10.1038/s41598-018-19660-z)
Supplement: Supplementary file 1 — Supplementary information [file 41598_2018_19660_MOESM1_ESM.pdf]

Deletion of DGCR8 in VSMCs of adult mice results in loss of vascular reactivity, reduced blood pressure and neointima formation

Short title: Role of DGCR8 in vascular smooth muscle cells of postnatal stage

Yanan Zou[1, 2], Zixuan Chen[2, 5] Brett L Jennings[3], Guannan Zhao[2], Qingqing Gu[2], Anindya Bhattacharya[4], Yan Cui[4], Bo Yu[1], Kafait U Malik[3], Junming Yue[2]

Figure legends

Figure S1. VSMC marker gene expression in aorta of DGCR8iKO and control mice.

DGCR8, SMA and SM22 expression in aorta of DGCR8iKO and control mice at day 21 and 28 were detected by Western blot

Figure S2. VSMC markers and cellular survival pathways in primary VSMCs of DGCR8 iKO and control mice

A: Phospho and total AKT pathways in primary VSMCs of DGCR8iKO and control mcie were detected by Western blot. **B:** phosphor and total ERK1/2 and VSMC markers in primary VSMCs of DGCR8iKO and control mice were detected by Western blot. **C.** VSMC marker MYH11 in primary VSMCs of DGCR8iKO and control mice was detected by Western blot.

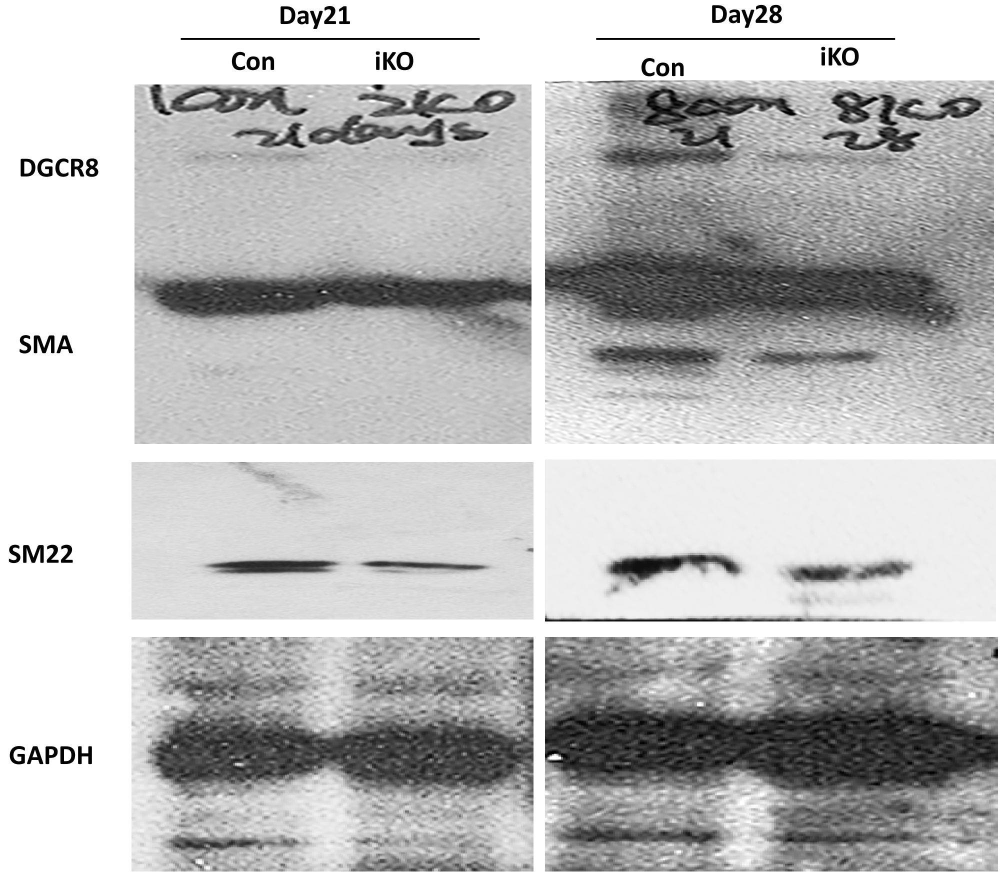
Day21
Day28
Con
iKO
Con
iKO
DGCR8
SMA
SM22
GAPDH

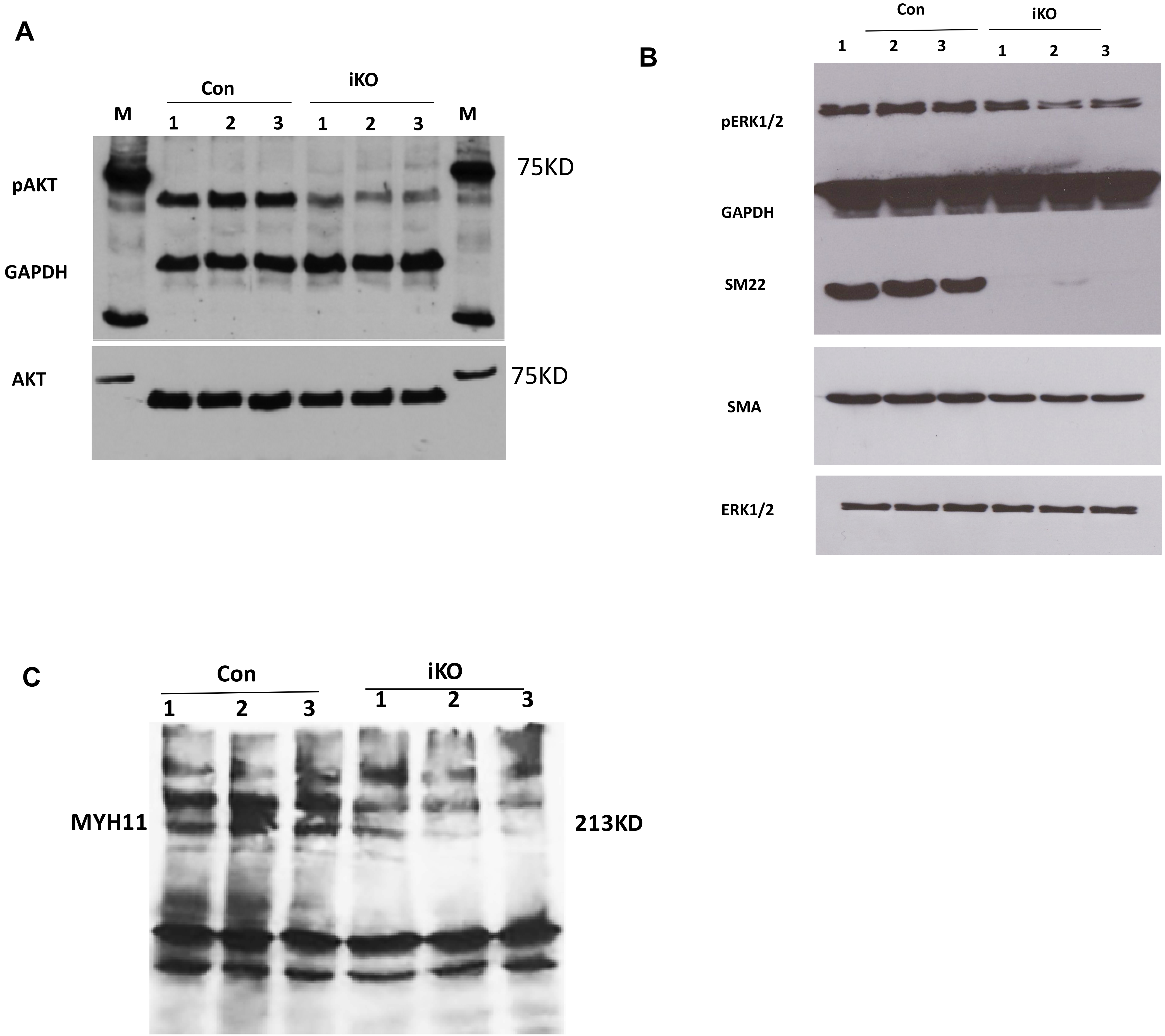
A
Con
iKO
M
1
2
3
1
2
3
M
pAKT
GAPDH
75KD
AKT
75KD
B
Con
iKO
1
2
3
1
2
3
pERK1/2
GAPDH
SM22
SMA
ERK1/2
C
Con
iKO
1
2
3
1
2
3
MYH11
213KD
